# Supplementary material for: Comparative study of the gut microbial community structure of Spodoptera frugiperda and Spodoptera literal (Lepidoptera)
Source: PeerJ. 2024 Jun 7;12:e17450. doi: 10.7717/peerj.17450 (PMC11164061; doi:10.7717/peerj.17450)
Supplement: Supplemental Information 1 — The circular plot consists of three layers: from inner to outer, the first layer represents the phylogenetic tree constructed from representative OTU sequences, with branch colors indicating the corresponding genus names, where each color represents a genus. The second layer shows the relative abundance distribution of OTUs, with the height of the bars representing the relative abundance of each OTU (transformed according to the minimum value due to the large range of relative abundance data). The third layer displays the confidence level distribution of OTU annotations, with the height of the bars indicating the confidence level of the annotations. [file peerj-12-17450-s001.pdf]

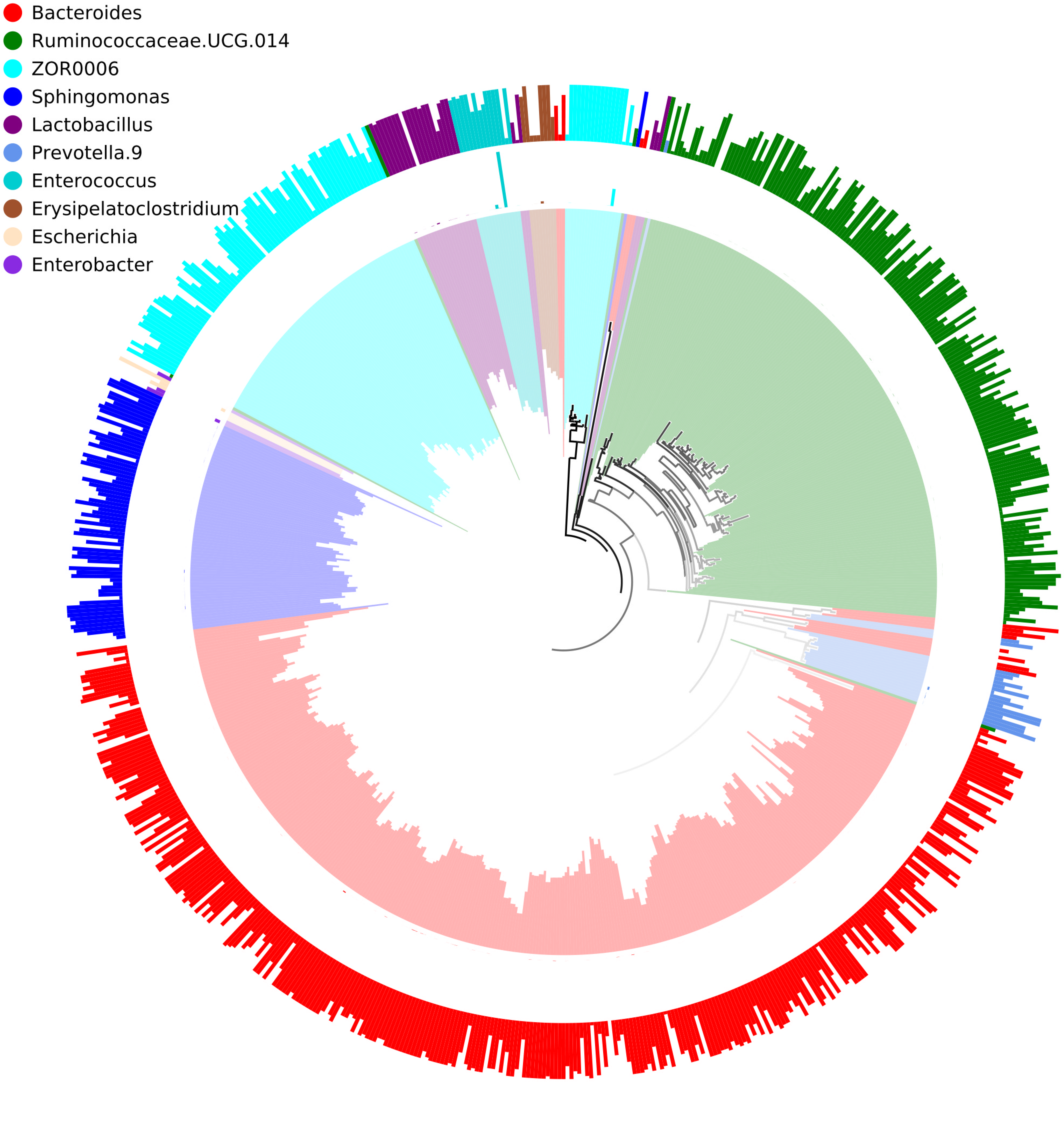
Bacteroides
Ruminococcaceae.UCG.014
ZOR0006
Sphingomonas
Lactobacillus
Prevotella.9
Enterococcus
Erysipelatoclostridium
Escherichia
Enterobacter
