# Supplementary material for: Comparative study of the gut microbial community structure of Spodoptera frugiperda and Spodoptera literal (Lepidoptera)
Source: PeerJ. 2024 Jun 7;12:e17450. doi: 10.7717/peerj.17450 (PMC11164061; doi:10.7717/peerj.17450)
Supplement: Supplemental Information 2 — ”*” represents a biomarker of midgut microbiota in S. litura and S.frugiperda [file peerj-12-17450-s002.doc]

Table 1 Biomarkers of midgut microbiota in S.litura and *S.frugiperda*

| Group | Phylum | Class | Order | Family | Genus |
| --- | --- | --- | --- | --- | --- |
| *S.litura* | Firmicutes | Erysipelotrichia | Erysipelostrichales | Erysipelostrichaceae | *Erysipelatoclostridium** |
|  | Firmicutes* |  |  |  |  |
|  | Proteobacteria | γ-proteobacteria | Enterobacteriales | Enterobacteriaceae | *Enterobacter** |
| *S.frugiperda* | Actinobacteria | Actinobacteria | Actinomycetales | Micrococcaceae* |  |
|  | Actinobacteria | Actinobacteria | Actinomycetales | Micrococcaceae | *Leucobacter** |
|  | Flavobacteria | Flavobacteria | Flavobacteriales | Flavobacteriaceae* |  |
|  | Proteobacteria | Alphaproteobacteri | Rhizobiales | Brucellaceae | *Pseudochrobactrum** |
|  | Actinobacteria | Actinobacteria | Bifidobacteriales | Bifidobacteriaceae | *Bifidobacterium** |
|  | Flavobacteria | Flavobacteria | Flavobacteriales* |  |  |
|  | Flavobacteria* |  |  |  |  |
|  | Actinobacteria | Actinobacteria | Bifidobacteriales* |  |  |
|  | Actinobacteria | Actinobacteria | Bifidobacteriales | Bifidobacteriaceae* |  |
|  | Actinobacteria | Actinobacteria | Actinomycetales | Microbacteriaceae* |  |
|  | Firmicutes | Bacillibacteria | Lactobacillales | Lactobacillaceae | *Lactobacillus** |
|  | Firmicutes | Bacillibacteria | Lactobacillales | Lactobacillaceae* |  |
|  | Proteobacteria | Alphaproteobacteri | Rhizobiales | Brucellaceae | *Ochrobactrum** |
|  | Firmicutes | Clostridia | Clostridiales | Peptostreptococcaceae* |  |
|  | Proteobacteria | Gammaproteobacteri | Enterobacteriale | Muribaculaceae* |  |
|  | Actinobacteria | Actinobacteria | Micrococcales* |  |  |
|  | Proteobacteria | Alphaproteobacteria | Rhizobiales | Brucellaceae* |  |
|  | Proteobacteria | γ-proteobacteria | Enterobacteriales | Enterobacteriaceae | *Escherichia** |
|  | Actinobacteria* |  |  |  |  |
|  | Proteobacteria | Alphaproteobacteri | Rhizobiales* |  |  |
|  | Bacteroidetes | Bacteroidia | Bacteroidales* |  |  |
|  | Bacteroidetes | Bacteroidia* |  |  |  |
|  | Firmicutes | Clostridia* |  |  |  |
|  | Firmicutes | Clostridia | Clostridiales* |  |  |
|  | Proteobacteria | Alphaproteobacteria* |  |  |  |
|  | Bacteroidetes* |  |  |  |  |
|  | Proteobacteria* |  |  |  |  |
|  | Firmicutes |  |  |  | *ZOR0006** |

"*" represents a biomarker of midgut microbiota in *S.litura* and *S.frugiperda*
